# Supplementary figures and images for: Inulin protects against the harmful effects of dietary emulsifiers on mice gut microbiome
Source: PeerJ. 2024 Mar 21;12:e17110. doi: 10.7717/peerj.17110 (PMC10961058; doi:10.7717/peerj.17110)

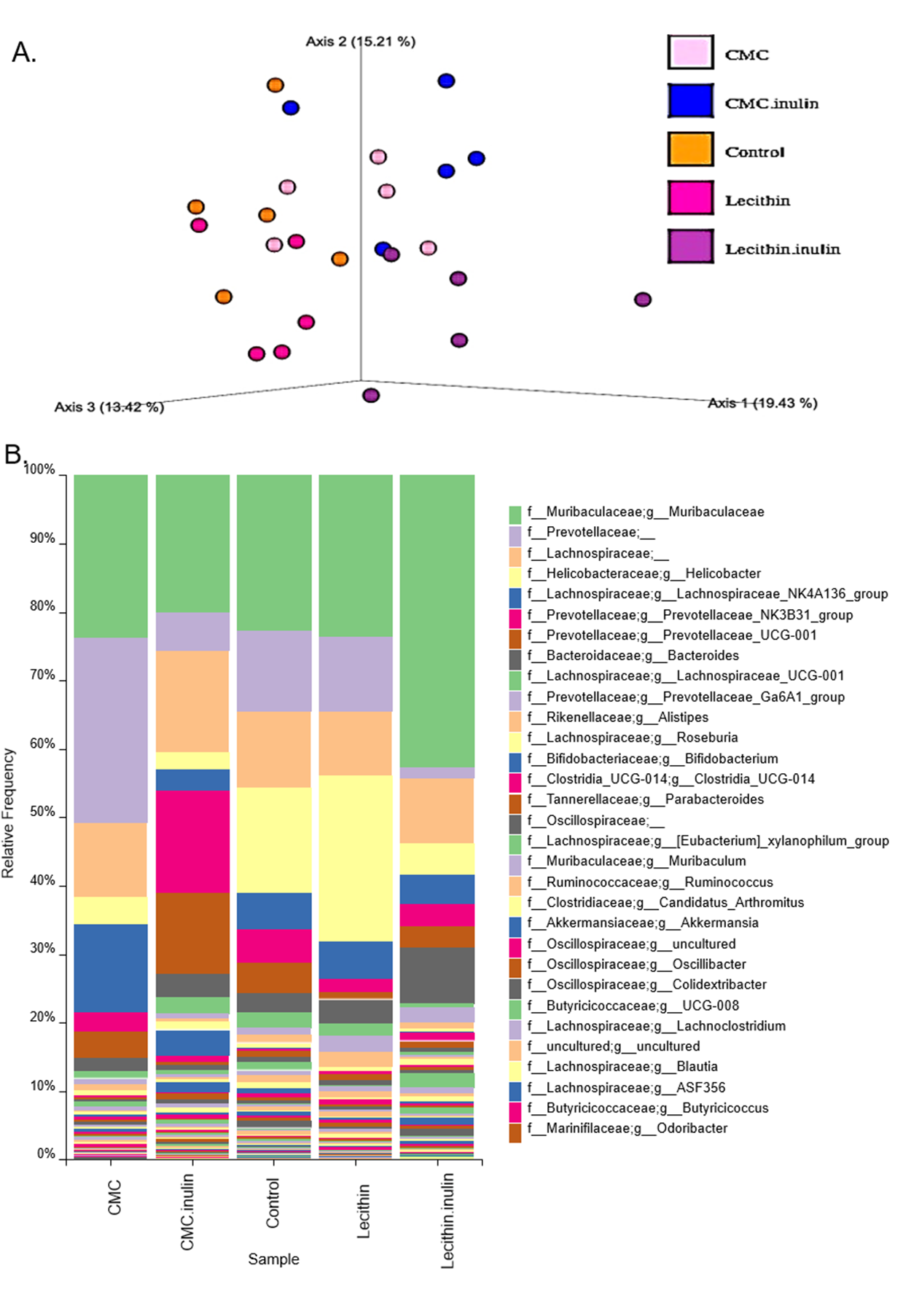

Supplement: Supplemental Information 1 — A) Unweighted UniFrac distances between groups (PERMANOVA, p < 0.005) as visualized by PCoA B) Family level taxonomic abundances across groups. [file peerj-12-17110-s001.png]

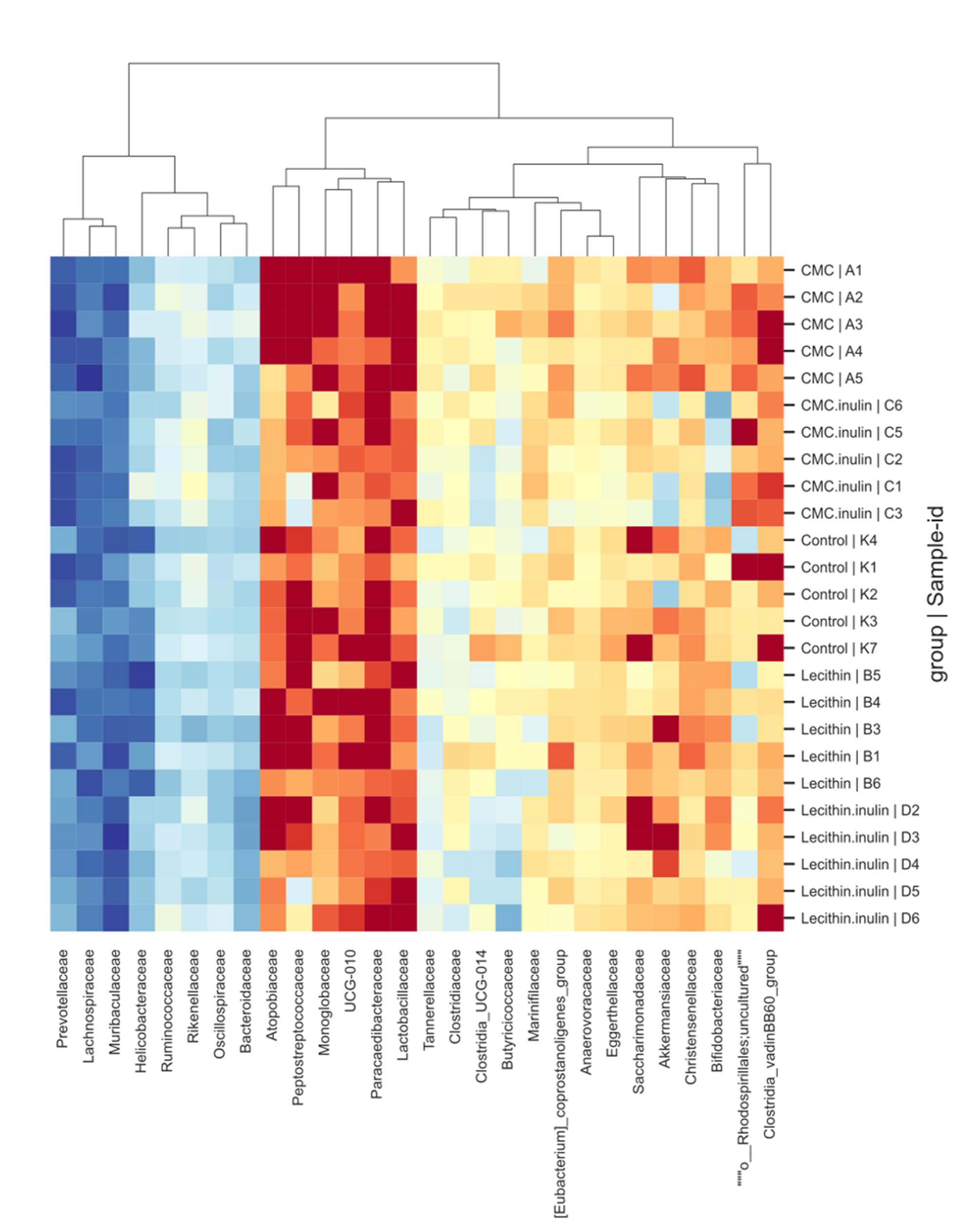

Supplement: Supplemental Information 2 — Group comparisons were performed by ANOVA and Kruskal-Wallis [file peerj-12-17110-s002.jpg]
