# Supplementary material for: Inulin protects against the harmful effects of dietary emulsifiers on mice gut microbiome
Source: PeerJ. 2024 Mar 21;12:e17110. doi: 10.7717/peerj.17110 (PMC10961058; doi:10.7717/peerj.17110)
Supplement: Supplemental Information 3 [file peerj-12-17110-s003.docx]

| Group 1 | Group 2 | pseudo-F | p-value | q-value |
| --- | --- | --- | --- | --- |
| CMC | CMC.inulin | 3.01 | 0.010 | **0.017** |
| CMC | Control | 2.893 | 0.012 | **0.017** |
| CMC | Lecithin | 2.494 | 0.012 | **0.017** |
| CMC | Lecithin.inulin | 3.568 | 0.012 | **0.017** |
| CMC+inulin | Control | 1.912 | 0.062 | 0.062 |
| CMC.inulin | Lecithin | 4 | 0.007 | **0.017** |
| CMC.inulin | Lecithin.inulin | 2.102 | 0.019 | **0.024** |
| Control | Lecithin | 1.677 | 0.039 | **0.043** |
| Control | Lecithin.inulin | 2.793 | 0.009 | **0.017** |
| Lecithin | Lecithin.inulin | 2.804 | 0.007 | **0.017** |
