# Supplementary material for: Inulin protects against the harmful effects of dietary emulsifiers on mice gut microbiome
Source: PeerJ. 2024 Mar 21;12:e17110. doi: 10.7717/peerj.17110 (PMC10961058; doi:10.7717/peerj.17110)
Supplement: Supplemental Information 4 — *Two fold or more ( ↑ / ↓ ) increase/decrease compared to control, ( ↗ / ↘ ) increase/decrease compared to CMC group, ( ↗ ↗ / ↘ ↘) increase/decrease compared to lecithin group. CMC: Carboxy methyl cellulose [file peerj-12-17110-s004.docx]

| Genus | Control  % | CMC  % | Lecithin  % | CMC-inulin  % | Lecithin-inulin  % |
| --- | --- | --- | --- | --- | --- |
| f__Prevotellaceae;g__ | 11.72 | 26.97 ↑ | 10.72 | 5.61 ↘ | 1.61↘↘ |
| g__Muribaculaceae | 22.85 | 23.80 | 23.77 | 20.07 | 42.75 ↗↗ |
| g__Lachnospiraceae_NK4A136_group | 5.39 | 12.92 ↑ | 5.57 | 3.06 ↘ | 4.24 |
| f__Lachnospiraceae;g__ | 11.18 | 10.97 | 9.51 | 14.88 | 9.55 |
| g__Helicobacter | 15.33 | 3.98 ↓ | 24.20 ↑ | 2.59 | 4.63 ↘↘ |
| g__Prevotellaceae_UCG-001 | 4.40 | 3.87 | 1.35 ↓ | 11.88 ↗ | 3.09 ↗↗ |
| g__Prevotellaceae_NK3B31_group | 4.73 | 2.60 ↓ | 1.71 ↓ | 14.75 ↗ | 3.04 ↗↗ |
| g__Bacteroides | 2.95 | 2.03 | 3.38 | 3.46 | 8.33 ↗↗ |
| g__Lachnospiraceae_UCG-001 | 2.27 | 1.19 ↓ | 1.76 ↓ | 2.51 ↗ | 0.66 ↘↘ |
| g__Alistipes | 1.28 | 0.85 | 2.17 | 0.36 ↘ | 1.04 ↘↘ |
| g__Roseburia | 0.67 | 0.77 | 0.76 | 1.38 | 0.37 |
| g__Prevotellaceae_Ga6A1_group | 1.01 | 0.77 | 2.28 ↑ | 0.70 | 2.07 |
| g__[Eubacterium]_xylanophilum_group | 1.33 | 0.72 | 0.13 ↓ | 0.56 | 0.39 ↗↗ |
| g__Muribaculum | 0.57 | 0.63 | 0.72 | 0.48 | 0.34 ↘↘ |
| f__Oscillospiraceae;g__uncultured | 0.49 | 0.48 | 0.73 | 0.22 ↘ | 0.32 ↘↘ |
| g__ASF356 | 0.51 | 0.46 | 0.27 | 0.31 | 0.30 |
| g__Marvinbryantia | 0.10 | 0.44 ↑ | 0.19 | 0.31 | 0.15 |
| f__Ruminococcaceae;g__ | 0.23 | 0.42 | 0.31 | 0.45 | 0.21 |
| g__Colidextribacter | 0.49 | 0.40 | 0.43 | 0.49 | 0.44 |
| f__Oscillospiraceae;g__ | 0.75 | 0.39 | 0.79 | 0.65 | 0.56 |
| g__Oscillibacter | 0.48 | 0.37 | 0.25 | 0.87 ↗ | 0.37 |
| g__Candidatus_Arthromitus | 0.88 | 0.37 ↓ | 0.33 ↓ | 0.36 | 0.80 ↗↗ |
| g__Paraprevotella | 0.17 | 0.31 ↑ | 0.58 ↑ | 0.21 | 0.14 ↘↘ |
| g__Anaerotruncus | 0.54 | 0.29 | 0.31 | 0.26 | 0.22 |
| g__Odoribacter | 0.45 | 0.29 | 0.59 | 0.11 ↘ | 0.26 ↘↘ |
| g__Parabacteroides | 0.81 | 0.28 ↓ | 0.82 | 0.45 | 0.89 |
| g__Blautia | 0.20 | 0.28 | 0.10 | 0.74 ↗ | 0.73 ↗↗ |
| g__Butyricicoccus | 0.34 | 0.25 | 0.38 | 0.56 ↗ | 0.26 |
| g__Akkermansia | 0.87 | 0.24 ↓ | 0.08 ↓ | 1.49 ↗ | 0.04 |
| g__Clostridia_UCG-014 | 0.30 | 0.22 | 0.41 | 1.11 ↗ | 1.34 ↗↗ |

Table S2. Genus level relative frequency distribution comparison between groups (30 most common features)

*Two fold or more (↑/↓) increase/decrease compared to control, (↗/↘) increase/decrease compared to CMC group, (↗↗/↘↘) increase/decrease compared to lecithin group. CMC: Carboxy methyl cellulose
